# Projected impact of diabetes on the Costa Rican healthcare system

**DOI:** 10.1186/s12939-020-01291-4

**Published:** 2020-10-26

**Authors:** Carolina Santamaría-Ulloa, Melina Montero-López

**Affiliations:** Instituto de Investigaciones en Salud, Universidad de Costa Rica, San José, Costa Rica

## Abstract

**Introduction:**

Costa Rica, similar to many other Latin American countries is undergoing population aging at a fast pace. As a result of the epidemiological transition, the prevalence of diabetes has increased. This condition impacts not only individual lives, but also the healthcare system. The goal of this study is to examine the expected impact of diabetes, in terms of economic costs on the healthcare system and lives lost. We will also project how long it will take for the number of elderly individuals who are diabetic to double in Costa Rica.

**Methods:**

CRELES (Costa Rican Longevity and Healthy Aging Study), a three-wave nationally representative longitudinal study, is the main source of data for this research (*n* = 2827). The projected impact of diabetes was estimated in three ways: length of time for the number of elderly individuals with diabetes to double; projected economic costs of diabetes-related hospitalizations and outpatient care; and years of life lost to diabetes at age 60. Data analyses and estimations used multiple regression models, longitudinal regression models, and Lee-Carter stochastic population projections.

**Results:**

Doubling time of the diabetic elderly population is projected to occur in 13 calendar years. This will cause increases in hospitalization and outpatient consultation costs. The impact of diabetes on life expectancy at age 60 around the year 2035 is estimated to lead to a loss of about 7 months of life. The rapid pace at which the absolute number of elderly people with diabetes will double is projected to result in a negative economic impact on the healthcare system. Lives will also be lost due to diabetes.

**Conclusion:**

Population aging will inevitably lead to an increasing number of elderly individuals, who are at greater risk for diabetes due to their lifelong exposure to risk factors. Actions to increase the quality of life of diabetic elderly are warranted. Decreasing the burden of diabetes on elderly populations and the Costa Rican healthcare system are necessary to impact the quantity and quality of life of incoming cohorts. Health promotion and prevention strategies that reduce diabetes risk factors are needed to improve the health of elderly populations.

## Introduction

Diabetes mellitus is a chronic disorder of carbohydrate, fat, and protein metabolism characterized by hyperglycemia resulting from defects in insulin secretion, insulin action, or both. There are two main forms of diabetes. Type 1 diabetes is due to an absolute insulin deficiency that requires patients to take exogenous insulin for survival. Its frequency is low relative to type 2 diabetes, which accounts for over 90% of cases globally. Type 2 diabetes (DM2) is characterized by insulin resistance and abnormal insulin secretion leading to relative rather than absolute insulin deficiency. Overweight and obesity may lead to insulin resistance, which, given genetic susceptibility may in turn lead to type 2 diabetes [35]. Once diagnosed, diabetes patients will have this condition for a lifetime, therefore lifestyle changes including diet and physical activity are usually prescribed. About two-thirds of elderly individuals with type 2 diabetes are treated with oral antihyperglycemic medications [49]. Patients with type 2 diabetes are not dependent on exogenous insulin, although some of them may require it to control their condition [59]. Diabetes mellitus type 2, which is the condition of interest for this study, is considered a public health issue. It is one of the most prevalent conditions and one of the leading causes of death worldwide. In 1980 there was an average of 108 million diabetic people in the world. By 2014 the number was 422 million [57]. Similar to other chronic diseases, diabetes prevalence is higher in low- and middle-income countries. Metabolic conditions, hypertension and diabetes included, have common risk factors, such as obesity. Although subjacent genetic factors play a role, environmental risk factors have been recognized as the most important determinants. Latin America, as a region, has adopted health and nutrition profiles of developed countries, which has contributed to the rise of obesity [1].

Costa Rica is a small Central American country that has achieved outstanding health standards. The current population is approximately 5.1 million, and 13% of Costa Ricans are aged 60 or older [12]. Total life expectancy in Costa Rica is 80 years, higher than life expectancy in the US, which is 78 years. This is despite Costa Rica having a per capita gross national income (GNI) of less than one-fourth that of the USA [34].

Costa Rican medicine is highly socialized. The country has a subsidized healthcare system established in 1941 as part of a government initiative. Since that time, the cost of public healthcare services has been largely paid by the government [41]. In the present day, more than half a century later, Costa Rica’s health policies have improved access to care through public services and universal social health insurance [53]. Government-funded health care is a key determinant of the country’s impressive health indicators. The high life expectancy in Costa Rica has been attributed to the funneling of resources towards education, to a strong primary care focus in the healthcare system, and to the role that a national health insurance fund has had in reducing economic barriers to healthcare access [10]. According to [42], the high life expectancy of Costa Rican adults is driven by the low incidence of conditions such as cardiovascular diseases, lung cancer and breast cancer. Nonetheless, Costa Ricans have a high incidence of diabetes mellitus, stomach cancer and, cervical cancer.

As a result of the demographic and epidemiological transitions in Costa Rica, the causes of morbidity and mortality have shifted from communicable to non-communicable diseases [40]. There has been an increase in diabetes incidence as early as the age of 30, and an important upsurge in the prevalence of diabetes in older adults [17]. Among the Costa Rican elderly, the prevalence is higher at younger ages and decreases with age [45]. This holds true not only for Costa Rica but also for Latin America at large.

Costa Rica, along with other Latin American countries, is in the middle of a diabetes and obesity epidemic which has partially resulted from sedentary habits and a westernized diet. Levels of self-reported diabetes and obesity in Latin America and the Caribbean (LAC) have been found to be as high or even higher than in the USA. In Costa Rica, 6 out of 10 adults are currently overweight or obese. Among the elderly, the prevalence of overweight and obesity is as high as in the younger adult population, with a greater impact on women [32].

Diabetes is currently the fourth cause of death in the elderly and has been increasing for the last three decades in Costa Rica [12]. It is also a well-established risk factor for coronary heart disease (CHD) [21], as well as for cardiovascular and cerebrovascular diseases [3]. Furthermore, hypertension and kidney disease are more prevalent in the diabetic population.

**Fig. 1:**
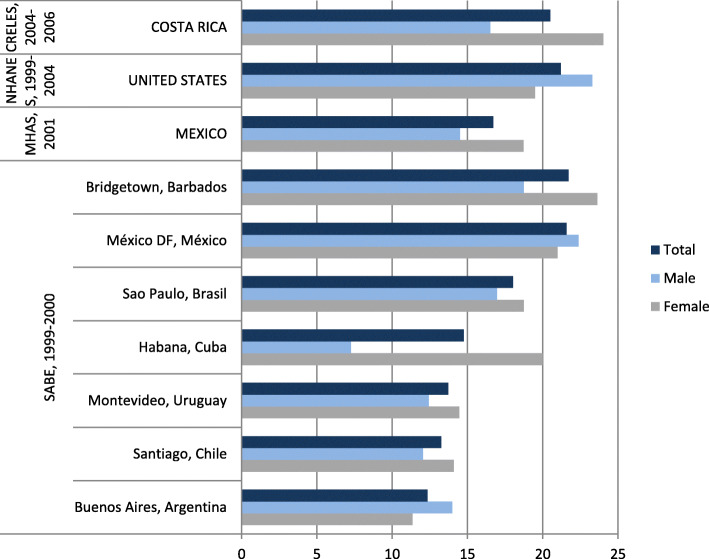
Prevalence of diabetes in the elderly population by sex. Selected countries and cities (percentages). Source: Own elaboration based on results from Brenes-Camacho and Rosero-Bixby [8]; McDonald [29]; Andrade [2]

A comparison of diabetes prevalence in Costa Rica, the United States, Mexico, and seven LAC cities is presented in Fig. 1. Although these figures are not strictly comparable in terms of the age of the reference population, or the year the survey was conducted, they all refer to elderly populations and were collected during similar time periods. As Fig. 1 shows, the prevalence in Costa Rica is one of the highest in the region, and is also higher among women in most cities and countries.

LAC countries have generated public policies in an effort to reduce the prevalence of diabetes, including added taxes for sugary drinks. Studies have shown that taxing these products significantly decreases their consumption and promotes the consumption of less unhealthy drinks [37]. Colchero, Popkin and Wen [15] showed that in Mexico, 1 year after the implementation of an excise tax on sugary drinks, purchases fell 6–12%, and purchases of less unhealthy drinks increased in 4%. No tax policies on sugary drinks are currently in place in Costa Rica but, since 2013, regulations applied to school canteens were implemented to reduce the consumption of sugary drinks and processed foods in public schools [31].

The diabetes epidemic has human and economic costs. Some of the costs come from premature mortality and from the burden on the healthcare system which is exacerbated by morbidity associated with diabetes complications. It must be acknowledged, however, that the burden of diabetes on health systems reflects only a fraction of the financial burden incurred by individuals with diabetes, their families and communities. Diabetes imposes costs on society in terms of lower returns on education, decreased income, lost productivity from job absenteeism and mortality, premature retirement and unemployment, and higher dependence on welfare [50, 58].

Diabetes is associated with higher comorbidity and with premature mortality which lead to a loss of both healthy life years and total life years. A study in Colombia conducted during 2006 and 2012 [20] sought to analyze the loss of healthy life years in a Colombian population. They found that among the population with chronic conditions women had a greater loss of healthy life years as compared to men. In other contexts outside Latin America, such as in Finland, it has also been found that diabetes has a negative effect on health-related-quality-of-life (HRQoL). Schanner, Falck, Keskitalo and Hautala [46] found that longer diabetes duration time was associated with a higher quality of life loss.

Little is known about the prevalence of diabetes and the determinants of this condition among elderly populations in Latin America because most epidemiologic studies have focused on the general population or younger population segments [4]. The objective of this study is to project the expected impact of diabetes in terms of doubling time of the diabetic elderly population, economic costs on the healthcare system, and lives lost. The expected impact of diabetes has not been quantified thus far. An estimation of the burden of this condition in the years to come will be an important input for the establishment of public policies that are relevant not only to Costa Rica, but also to other developing countries facing similar scenarios in Latin America.

## Methods

CRELES (Costa Rican Longevity and Healthy Aging Study), a three-wave nationally representative longitudinal study, is the main source of data for this research (*n* = 2827). The baseline interview was conducted between 2004 and 2006, the second wave between 2006 and 2007, and the third wave between 2008 and 2009. Mortality was tracked up to October 31, 2017, by linking the CRELES dataset with the National Death Registry. More details on this survey have been previously published [44, 45].

Self-reports were used to define the diabetes status of individuals. The corresponding items in the questionnaire were: “Has a medical doctor ever told you that you have diabetes or high blood sugar levels?” and “How old were you when you were first told that you had diabetes”. To avoid the potential inclusion of individuals with type 1 diabetes, only individuals reporting a diagnosis at 30 years or older were classified as having the condition. This criterion has been used in other population studies [22, 23, 45, 49].

Sociodemographic variables included in regression models were education, income, sex and age. Education was a dichotomous variable for incomplete/complete primary school; complete primary was defined as six or more years of formal education. Income was a low/high dichotomous variable, with a cut-off point of 50,000 colones (Costa Rican currency) per individual per month. This was equivalent to approximately 100 United States Dollars (USD) during the wave 1 time period (2004–2006). Income was defined as the older person’s own income, if not married, or the couple’s mean monthly income, if married. This income cut-off has been used in similar studies with CRELES [8, 30].

Risk factors included in regression models were body mass index (BMI) and waist circumference, which are indicators of general and abdominal obesity, respectively. As for BMI, individuals were classified as underweight (< 18.5 kg/m^2^), normal weight (18.5–24.9), overweight (25.0–29.9), or obese (≥30.0 kg/m^2^) [56]. Waist circumference categories for men were normal (< 94 cm), increased risk of metabolic complications (94–101), and substantially increased risk of metabolic complications (≥102 cm). Waist circumference categories for women were normal (< 80 cm), increased risk of metabolic complications (80–87), and substantially increased risk of metabolic complications (≥88 cm) [56].

Behavioral health risks included in analyses were smoking, alcohol consumption, total daily energy intake, and regular physical activity. Individuals living with a smoking partner were classified as passive smokers, when they were not active smokers themselves. Smoking behavior refers to 100 or more cigarettes or cigars during participants’ lives. Categories were defined as never smoked, former passive or active smoker, current passive smoker, and current active smoker. Alcohol consumption refers to alcoholic beverages ever consumed during individuals’ lives. Categories were defined as never consumed alcohol, former consumer, and current alcohol consumer.

Total daily energy intake was estimated from a 10-min Food Frequency Questionnaire, with a 3000 kcal/day cut-off. This standard cut point is associated with differential risk of cardiovascular disease [9] and has been used in similar population studies [30, 38, 43]. Regular physical activity was defined as three or more days per week of exercise routines or other physical rigorous activities like sports, jogging, dancing, or heavy work during the 12 months preceding the baseline interview.

Geriatric syndromes included in the models were polypharmacy, functional dependency and geriatric depression. Polypharmacy was defined as 5 or more medications daily. Functional dependency was defined as limitations in 7 or more of the 14 activities of daily living (ADL) and the instrumental activities of daily living (IADL). ADL were crossing the bedroom from side to side, bathing, self-feeding, going to bed, toileting, nail trimming, walking, climbing stairs, pushing objects, and raising arms. IADL were cooking, handling money, shopping, and taking medications. The Geriatric Depression Scale (Short Form) which includes 15 items [47] was used to classify participants according to their depression level. Normal was defined as less than 6 depression symptoms; mild depression as 6 to 9 and depression as 10 to 15 depression symptoms.

Health condition variables included in the mortality analyses were previous diagnoses of diabetes, hypertension, dyslipidemia, cardiovascular disease, cancer, and lung disease. Blood pressure was measured twice during the interview, yielding two measures of systolic pressure and two of diastolic pressure. Following Méndez-Chacón et al. [30] individuals were classified as hypertensive if they either had a previous medical diagnosis; or had blood pressure of 140/90 or higher in three out of the four systolic and diastolic measures during the interview; or if they were taking antihypertensive medications.

Dyslipidemia was defined as having at least one of hypercholesterolemia or hypertriglyceridemia. Hypercholesterolemia was defined as Total/HDL ratio of 5.92 or greater. Hypertriglyceridemia was defined as 150 mg/dl or greater. Individuals who were not fasting when their blood sample was taken have missing information on these biomarkers. Cardiovascular disease was defined as the diagnosis of at least one of myocardial infarction, ischemic heart disease without infarction, or stroke.

Data analyses and estimations were conducted with STATA computer software [52]. The analyses include descriptive statistics, multiple regression models, longitudinal regression models, and Lee-Carter stochastic population projections.

### Projection of diabetes prevalence in the elderly

To project the expected size of this diabetic elderly population, we first estimated its rates of prevalence, incidence and mortality. The main input for prevalence projections was the incidence and mortality rates computed for 5-year age groups ending in the open 95+ group. In the estimations of the prevalence of diabetes in the elderly, migration was not taken into consideration; only incidence and mortality were allowed to affect prevalence projections. These projections are therefore under the assumptions of null migration and constant incidence and mortality along time.

The size of the diabetic population was computed 5 years back from the year 2006 to the year 2001, and then 5 years back again from the year 2001 to 1996. Growth rates between these time-points were used to project the size of the diabetic population assuming linear growth. Projections were estimated with the following formula:

$$ {{\mathrm{N}}_{\mathrm{x}}}^{\mathrm{d}}=\left[{{\mathrm{N}}_{\mathrm{x}+\mathrm{t}}}^{\mathrm{d}}-\left({\mathrm{N}}_{\mathrm{x}}{\ast}_{\mathrm{t}}{\mathrm{d}}_{\mathrm{x}}\right)\right]/\left(1{-}_{\mathrm{t}}{\mathrm{d}}_{\mathrm{x}}{\ast}_{\mathrm{t}}{\mathrm{q}}_{\mathrm{x}}\right) $$

where:

*N*_*x:*_ Total population at age x

*N*_*x*_^*d*^: Diabetic population at age x

*N*_*x*_^*nd*^: Non-diabetic population at age x

_*t*_*d*_*x*_: Diabetes incidence rate for the population aged x to x + t

_*t*_*q*_*x*_^*d*^: Probability of dying for the diabetic population aged x to x + t

Diabetic population size for each age-group (N_x_^d^) was estimated based on age-specific prevalence rates and the official total population size (N_x_) in 2006 [12]. Prevalence rates for the elderly (60+) are this study’s computations from multiple regression models. For the younger adult population (30–59) prevalence rates are the five-year age-group national estimates reported by another study in Costa Rica [39].

Incidence rates in the above formulas (_t_d_x_) are this study’s computations from longitudinal regression models for ages 30+ using the respective information on the age of diagnosis (and assuming the absence of selection survival). Death rates for the diabetic population (_t_m_x_^d^) for ages 30–59 are assumed to be the same as all-cause mortality in the general population from the National Death Registry [12]. Death rates for the diabetic population for ages 60+ are this study’s estimates from longitudinal regression models of all-cause mortality in the diabetic elderly. The age-specific probabilities of dying for the diabetic population (_t_q_x_) were derived from the _t_m_x_^d^ using the relations and separation factors in Coale and Demeny [14] West model life tables.

Based on our population projections, the doubling time of the diabetic elderly population was estimated. Doubling time refers to the number of years it takes for the diabetic elderly population to double in size in Costa Rica under the assumption of a constant linear growth rate. Doubling time of the diabetic elderly population was also estimated for six hypothetical scenarios. Each scenario was under the assumptions of a linear population growth and a constant age-pattern of the incidence as observed in CRELES. For each scenario age-specific incidence rates were allowed to increase or decrease by 25, 50, and 75%, respectively.

### Impact of diabetes on future healthcare costs

Future costs of health care for the elderly population were estimated using this study projected prevalence of diabetes and previously published estimations of current individual use of healthcare services [45]. Hospitalizations and outpatient consultations were modeled using two-part models, a common approach in health economics [18]. Costs were recorded based on the mean volume of utilization of hospitalizations and outpatient visits over a calendar year to avoid bias due to seasonality in the patterns of use of these services.

The costs of health care services used in this research are from the provider’s perspective, reported by the Costa Rican Social Security Fund (CCSS, for its Spanish acronym). Monthly current costs are publicly available at http://www.ccss.sa.cr. These monthly costs for each health care service are estimated by the CCSS as the total expenses incurred in one specific service divided by the total production in the same service over 1 month. Costs are reported in this study in United States Dollars from the year 2011 (2011 USD). We assumed that current patterns of healthcare services utilization remain constant in the future both in the diabetic as in the non-diabetic elderly.

### Impact of diabetes on future life expectancy

The Lee-Carter method for mortality forecasting was used to forecast all-cause-mortality in this study. The Lee-Carter (LC) procedure is a stochastic model. It, therefore, allows for the quantification of uncertainty in the estimates. This method has been widely adopted in the USA, and it has also been used in the G7 countries and Australia [5]. Modifications and extensions have also been proposed to the method [6, 28].

Historical mortality rates for the 70s decade was not used in the time-series for mortality forecasting purposes. A significant decrease in infant mortality occurred during the 60s and 70s in Costa Rica. Using the 70s-decade mortality rates as an input for the estimation would have implied that such drastic mortality declines would have a chance to repeat in the future, which is not reasonable. Historical data on mortality from 1980 to 2010 was used to forecast 25 years of mortality up to the year 2035. Forecasts were estimated for (1) all-cause mortality and for (2) non-diabetes-related mortality. Forecasts were estimated using the LCFIT software [51], which produces a set of forecasted rates as an output. Median mortality rates by age-group and calendar year, as well as their corresponding confidence intervals, were estimated from the set of forecasted rates.

Using forecasted mortality rates to estimate life tables, two approaches were used to project the impact of diabetes in terms of life lost. They were based on estimations of life expectancy at birth (e_0_) and life expectancy at age 60 (e_60_).

In our first approach, we estimated e_0_ and one hypothetical scenario. We forecasted the total life expectancy at birth, and the total life expectancy at birth that would result from all mortality causes except diabetes.

In our second approach, we estimated e_60_, a commonly used indicator of longevity. We forecasted the total life expectancy at age 60, and two hypothetical scenarios. Our first hypothetical e_60_ scenario was the total e_60_ that would result from all mortality causes except diabetes. Our second hypothetical e_60_ scenario was the total e_60_ that would result from removing diabetes mortality and adding diabetes-caused mortality from a longitudinal competing-risks model. In this competing-risks model, the mortality hazard was computed as a function of diabetes-caused mortality, and the competing event was mortality due to any other cause. Forecasts were estimated with the assumption that the observed pattern and level of diabetes-caused mortality remains constant in the future.

Years of life lost to diabetes were estimated as the difference between the total life expectancy at age 60, minus the life expectancy at age 60 that would result from removing diabetes mortality and adding diabetes-caused mortality from the longitudinal competing risks model.

## Results

As shown in Table 1, diabetes prevalence is 21% among the Costa Rican elderly population. The proportion of females is significantly higher among the diabetic as compared to the non-diabetic (61% vs. 50%, *p* < 0.01) population. More than half of the Costa Rican elderly are in the younger 60 to 69 age group. The oldest old, aged 80+ have a share of 10% among the diabetic elderly as compared to 16% among the non-diabetic (*p* < 0.01).

**Table 1:** Descriptive information of the CRELES Costa Rican elderly at baseline: 2004–2006 (weighted estimates)

Characteristic, ***n = 2827 unless otherwise noted***	Elderly population^a^		
	Total	Non- diabetic	Diabetic
***Sociodemographics***			
*Education: % with complete primary,*	49,0	49,6	46,8
*Low income, 2799*	40,6	40,7	40,2
*Sex*			
Male	47,5	49,8	38,8***
Female	52,5	50,3	61,2***
*Age*			
60–69 yrs	53,8	53,5	54,7**
70–79 yrs	31,6	30,6	35,3***
80+	14,7	15,9	10,0***
***Risk factors***			
*Waist circumference, 2632*			
Normal	31,7	35,7	16,7***
Increased risk of metabolic complications	23,2	23,7	21,3
Substantially increased risk of metabolic complications	45,1	40,7	62,0***
*Body Mass Index, 2698*			
Underweight	3,3	4,0	0,6***
Normal	28,5	31,4	17,2***
Overweight	42,1	42,1	42,2**
Obese	26,1	22,5	40,0***
***Behavioral health risks***			
*Smoking, 2810*			
Never	35,7	36,0	34,7
Former active or passive smoker	38,9	39,9	35,2**
Current passive smoker	13,8	13,1	16,5*
Current active smoker	11,6	11,0	13,7*
*Alcohol,2809*			
Never	34,5	34,5	34,6
Former alcohol drinker	29,7	30,9	24,9***
Current alcohol drinker	35,8	34,6	40,4***
*Calorie daily consumption > =3000, 2819*	12,3	13,0	9,8
*Regular physical activity*	31,3	33,8	22,0***
***Geriatric Syndromes***			
*Polypharmacy 2219*	34,1	27,1	53,7***
*Functional dependency, 2822*	11,8	11,6	12,7
*Geriatric depression, 1769*			
Normal	81,9	83,2	77,3*
Mildly depressed	11,8	11,1	14,3
Depressed	6,3	5,7	8,3*
***Mean cost of health care (2011 USD)***			
*Cost of hospitalization*	827	745	1124**
Age			
60–69 yrs	570	493	841**
70–79 yrs	952	852	1280**
80+	1634	1503	2424
*Cost of outpatient care*	337	319	404**
Age			
60–69 yrs	328	308	398**
70–79 yrs	347	328	409**
80+	355	343	427**
***Health condition***			
Chronic morbidity			
Hypertension, *2823*	64,5	60,0	81,8***
*Dyslipidemia*^*b*^*, 2656*	51,2	50,2	55,3***
Elevated Total/HDL cholesterol ratio, *2654*	28,5	27,8	30,8*
Elevated triglycerides, 2573	44,9	44,2	47,6***
*Cardiovascular disease*			
Myocardial infarction	4,6	3,7	7,9***
Ischemic heart attack (no infarction)	12,0	10,9	16,4**
Stroke	3,8	3,4	5,7*
Cancer	5,8	5,2	8,0
Lung disease	16,6	15,6	20,5**

Significance levels: ***: *p* < 0.01, **: *p* < 0.05, *: *p* < 0.10

^a^Chi-square tests were used for non-diabetic vs. diabetic stratum comparisons of categorical variables. T-tests were used for continuous variables

^b^Dyslipidemia refers to any or both: hypercholesterolemia (Total/HDL ratio) and hypertriglyceridemia

In general terms, risk factors and behaviors are different in the diabetic and the non-diabetic elderly population. The prevalence of central and general obesity as measured by waist circumference and body mass index is significantly higher among diabetic individuals (62% vs. 41, and 40% vs. 22%, *p* < 0.01). The prevalence of regular physical activity is significantly lower among the diabetic elderly (22% vs. 34%, *p* < 0.01). This physical activity measure includes both individuals who being able to exercise do not do so, and those whose health condition prevents beneficial physical activities. Among the diabetic elderly, hypertension is more prevalent (82% vs. 60%, p < 0.01). Myocardial infarction, ischemic heart attack and lung disease are also significantly higher among the diabetic (Table 1).

The mean costs of healthcare are significantly different between the diabetic and non-diabetic population. Hospitalization costs are significantly higher among diabetic elderly aged 60 to 79. Outpatient care costs are significantly higher for diabetic as compared to non-diabetic elderly population (Table 1).

### Doubling time of the diabetic elderly population

**Fig. 2:**
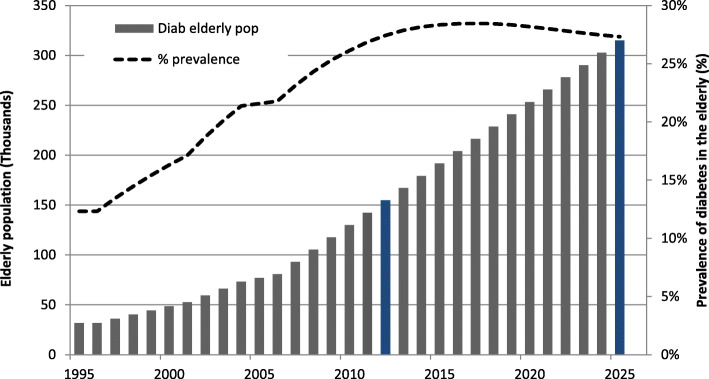
Projections of diabetic population size, and prevalence rate in the elderly. Costa Rica: 1996–2025 (Left y-axis refers to population (thousands), represented as solid bars. Right y-axis refers to the prevalence of diabetes in the elderly population (%), represented as a dotted line)

Using the information on diabetes prevalence at CRELES first wave, as well as 5-year-age-group incidence and mortality rates, the size of the diabetic population was estimated back from the year 2006 (the end of CRELES wave 1) to the years 2001 and 1996. As a result, mean annualized growth rates for the 1996–2001 and 2001–2006 periods were estimated under the assumption of linear growth of the elderly diabetic population. Diabetes prevalence was projected to reach a 27% rate in 2025. Doubling time of the diabetic elderly population is estimated to occur in 13 calendar years in this country (Fig. 2).

Considering population projections in this study are used to project healthcare costs into the future, a sensitivity analysis of the doubling time was conducted based on six different hypothetical scenarios, in which the incidence rates were allowed to increase or decrease by 25, 50, and 75%, respectively. The 13 years doubling time holds as a robust estimation (Table 2).

**Table 2:** Doubling time of the diabetic elderly population for hypothetical incidence level scenarios

Scenarios		Diabetic elderly population size in 2025	Prevalence in 2025 (%)	Doubling time (years)
**Worst case scenarios: Incidence rate increases**				
(1)	75% increase	372,870	30	12,0
(2)	50% increase	351,697	29	12,5
(3)	25% increase	332,472	28	12,5
**Counterfactual: Incidence rate remains constant**		**314,939**	**27**	**13,0**
**Best case scenarios: Incidence rate decreases**				
(4)	25% decrease	298,885	26	13,0
(5)	50% decrease	284,128	26	13,0
(6)	75% decrease	270,519	25	13,5

### Projected economic costs of healthcare

**Fig. 3:**
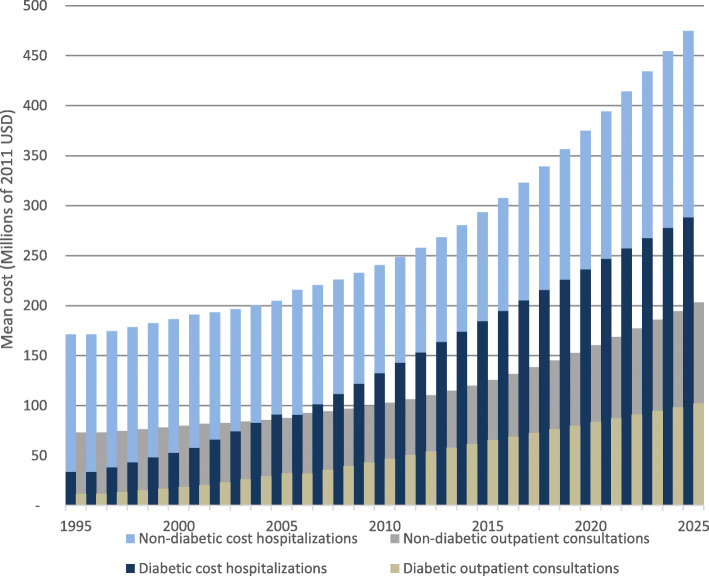
Projected annualized mean costs of hospitalization and outpatient consultations of the Costa Rican elderly population under the assumption of linear growth of diabetes prevalence (2011 USD)

The impact of diabetes prevalence on future healthcare costs was estimated based on the increase in total hospitalization and outpatient care costs. Hospitalization costs are higher than those of outpatient care. Costs associated with the hospitalization of diabetic elderly are projected to have a 38% share on total costs of hospitalizations in the elderly in the year 2025, which is 8 percentage points higher as compared to 20 years back in 2005 (Fig. 3). Outpatient consultations are projected to represent 34% of total consultation costs in the elderly in 2025, which is also 8 percentage points higher than in 2005 (Fig. 3).

### Years of life lost to diabetes

**Fig. 4:**
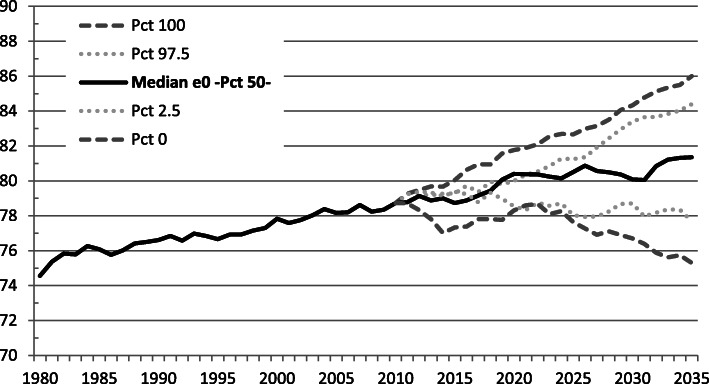
Forecast of total life expectancy at birth based on all-cause mortality. Costa Rica: 1980–2035

**Fig. 5:**
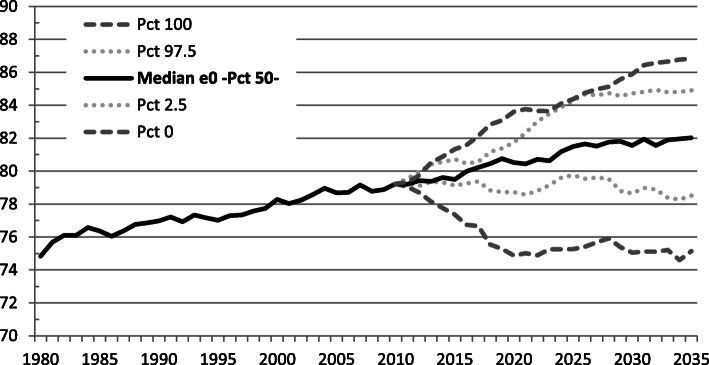
Forecast of hypothetical total life expectancy removing diabetes as a cause of death. Costa Rica: 1980–2035

The current total life expectancy at birth (e_0_) in Costa Rica is 80.07 years. Based on historical mortality, we forecast e_0_ to reach 81.35 years in 2035 (Fig. 4). If diabetes were not a direct cause of death for any individual, e_0_ would be higher. Under the hypothetical scenario of deleting diabetes as a direct cause of death for any individual aged 30+, we forecast e0 would reach 82.02 years in 2035 (Fig. 5).

When studying the impact of a specific disease on mortality, it is often assumed that one can subtract off the number of deaths caused by that specific disease. However, when studying cause-specific mortality, it is wiser to assume a counterfactual scenario, in which competing risks of death are computed [36]. Both approaches to the estimation of life expectancy at age 60 are shown in Table 3, as scenarios (2) and (3), respectively. Diabetes is removed as a cause of death in scenario (2). Whereas e_60_ estimation results from removing diabetes mortality and adding diabetes-caused mortality from a longitudinal competing risk model in scenario (3). Years of life lost to diabetes were estimated based on the latter scenario, which yields a finer estimation of the impact.

**Table 3:** Forecast of total life expectancy at age 60 (95% CI). Costa Rica: 2025 and 2035

Forecast	2025	2035
(1) *e*_60_ based on all-cause-mortality	24,33 (24,28-24,38)	24,97 (24,90-25,04)
(2) *e*_60_ deleting DM2 mortality as a cause of death	24,48 (24,79-24,89)	25,54 (25,47-25,61)
(3) *e*_60_ deleting DM2 mortaliy as a casue of death and adding DM2-caused mortality from a competing risks regresssion model	23,73 (23,68-23,77)	24,37 (24,30-24,43)
(1)–(3) Years of life lost to diabetes	0,602 (0,600-0,605)	0,597 (0,592-0,603)

We estimate a seven-months of life loss in 2025 as an impact of diabetes on e_60_. The same life loss estimation holds for 2035. These estimations and their corresponding confidence intervals are presented in Table 3.

## Discussion

Gender differences are found among the diabetic elderlies. Although the non-diabetic population is equally distributed among males and females, the diabetic population is heavily represented by females in elderly Costa Ricans. These gender differences have not been consistently found in the literature. Nonetheless, it has been hypothesized that these differences may come from a lower prevalence of obesity and from a late detection of the condition for males in this country [45].

Diabetes risk factors and behaviors are not homogenously distributed among the population. Because of their modifiable nature, they constitute a window of opportunity to modulate intermediate and final health outcomes. Obesity, which is the primary risk factor for this condition, is significantly more prevalent among the diabetic elderly. Had this risk factor been modified in previous stages of individuals’ life cycle, differences between diabetic and non-diabetic population would probably be smaller. Along the same lines, regular physical activity is less prevalent in this population, which may reflect a lifetime behavior.

Age differences found between diabetic and non-diabetic elderly in this study are a result of the diabetes associated premature mortality. Diabetes-caused mortality has a greater share of general mortality at younger ages. As it has also been shown in other studies, this condition is associated with increased risks of death from all causes [23], which translates into premature mortality.

Improving population health is the main goal for any health system. Our results show that diabetes leads to a loss of about 7 months on life expectancy at age 60. Life loss evidenced in this study is known to be preceded by a loss of quality of life. Previously published studies on health-related quality of life (HRQoL) in Finland, for example, have found that as compared to the non-diabetic population, HRQoL scores of the diabetic population are negatively affected especially as the duration of disease increases [46]. Studies on disability-adjusted life expectancy in Cuba, for example, found that diabetes decreased life expectancy approximately less than 1 year [19]. Another study of Cárdenas, [11] reported that diabetes impact on quality-of-life occurred mainly in elder low socioeconomic status women, with few support networks, and without healthcare insurance.

Taking into account the burden of DM2 both in terms of the impact on the individual and the costs it represents for the healthcare system, it is important to analyze alternatives to improve lifestyles. According to García, Camarelles, Muñoz, Gómez, Arangoe, Ramírez, and colleagues [16], some lifestyle changes like physical activity and healthy eating have shown to improve population health and decrease the burden of the disease by reducing its incidence and the complications associated to those who are already ill. These initiatives are important to be applied in all healthcare levels, but especially in the first level of care.

A deterministic forecast specifies time-changes over the forecast period in terms of deterministic (i.e., not random) assumptions. In the stochastic forecast, different from deterministic projections, the role of the forecaster’s subjective judgment is reduced [27] and the uncertainty in the estimates may be quantified. Based on a stochastic projection methodology, our study yielded a robust estimation of the diabetes prevalence projection and the doubling time of the diabetic elderly in Costa Rica. Using the same CRELES dataset, but a deterministic projection methodology, which was a variation of the cohort-component method, [7] attained remarkably similar results. According to our study, 27% of the elderly population is projected to be diabetic by 2025 in Costa Rica. Brenes [7] estimated a 28% prevalence of diabetes in the elderly for the same year. Our study estimates that the size of the diabetic elderly population will double in 13 years. Along the same line, [7] estimated it would take 25 years for Costa Rican diabetic elderly population size to quadruple. The main feature of a stochastic approach to forecast is that estimations can be accompanied by confidence intervals [25], as shown in this study.

Hypothetical scenarios of decreasing diabetes incidence rates would imply a great effort from a public health point of view. Still, as this study shows, the resulting doubling time would not dramatically change under different scenarios. Even for the most optimistic scenario of 75% decrease in the incidence rate, doubling time would be 13,5 calendar years, which is very close to the 13,0 years estimated for the counterfactual. Doubling time of diabetic elderly population is mainly driven by the population aging inertia.

Obesity, a risk factor for other chronic conditions, is associated with a significant burden of hypertension and myocardial infarction which not only translates into a lower life expectancy as shown in our results but also into a lower quality of life. A previous study has reported that 20 years after diagnosis of diabetes there is a 45% chance of heart failure and a 65% chance of retinopathy [24]. Diabetes is associated with long-term damage, dysfunction, and failure of organs, especially the eyes, kidneys, nerves, heart, and blood vessels. Diabetes also significantly increases the risk of stroke, chronic kidney disease, cancer and all-cause mortality [49]. For a Costa Rican urban population of adult diabetic patients, [26] have reported a 33.6% prevalence of nephropathy, 30.6% prevalence of neuropathy, and 24.8% prevalence of microproteinuria. This high prevalence of complications causes a diabetic population to make heavier use of outpatient consultations and hospitalizations, which adds to the burden to the healthcare system.

The prevalence of DM2 is a challenge for all countries in the region, because of its costs to the healthcare system and the high mortality it causes. In Costa Rica elderly diabetic patients are more inclined to be hospitalized and to make use of outpatient consultations [45]. As the diabetic elderly population increases, the total costs of hospitalizations and outpatient care will also increase, as shown in this study. A higher share of costs associated with diabetic elderly is projected to occur in the near future.

Gender and geographical inequalities in diabetes incidence, prevalence and mortality have been previously described in this elderly Costa Rican population [45]. Furthermore, this study describes existing inequalities such as higher obesity, lower regular physical activity, and higher incidence of myocardial infarction, ischemic heart attack and lung disease among diabetic individuals. Because Costa Rica has a universal and solidary healthcare system, higher expenditures associated with diabetes inequalities increase the burden on the healthcare system. This increased burden caused by diabetes in the elderly may also have an impact on the attention of other health conditions for the general population.

This increasing burden of diabetes has already been perceived by Costa Rican authorities. A research conducted in a Costa Rican population had shown that diabetic individuals made 1.55 more medical visits and had 1.98 times more hospitalizations than their non-diabetic peers [33]. The most frequent causes of consultation in the elderly (22%) are those related to the circulatory system, which include hypertension. The second most important causes (16%) are endocrine, nutritional and metabolism pathologies, which include diabetes [13]. These causes of morbidity, diabetes being one of the most relevant, represent high costs to the healthcare system.

Diabetes prevalence will continue to rise in the elderly. This increased prevalence is the main reason for the growing burden of diabetes not only in developing but also in developed countries [48]. Previous studies have shown that in individuals at high risk, a combination of weight loss, physical activity and dietary advice leads to a significant reduction in incidence. Prevention using lifestyle interventions as a strategy lead to higher cost-effective reductions in incidence among populations at high risk than strategies that use drugs for prevention [55].

Because it is also expected that a substantial proportion of diabetes will arise in individuals not identified as being at high risk, broader strategies are also necessary. These strategies include public policy to modify the obesogenic environment in which populations live [54]. It becomes necessary to generate public policies with actions focused on promoting health that allow the population to have equitable access to health information, spaces for physical activity and healthy eating. Policies should be adapted to different age groups and populations, understanding that early-stage actions influence life cycle behaviors. Local support is important for policy implementation because the local level is responsible for operationalization of actions, and the local actors are the ones who know better which populations are disadvantaged.

Because of the use of self-reported measures, information bias is a limitation of this study, which might result in an under estimation of the prevalence of diabetes. Selection bias is also a limitation. Reported results are susceptible to selection bias because they rely on the population that survived at least to the age of 60 in 2005, when the baseline survey was conducted. Another limitation of this study is the impossibility of ascertaining birth cohort effects. Observed diabetes patterns may result from cohort effects that have occurred over time. It may be hypothesized for example, that individuals from older cohorts might have been less exposed to risk factors such as physical inactivity or high fat diets than individuals from younger cohorts who are now experiencing higher rates of diabetes prevalence. In this study, the same participants recruited for baseline, were re-interviewed for the second and third waves. Different from some panel studies, cohorts of individuals were not therefore renewed within each wave in this study. As a result, a large enough sample size of individuals within cohorts is not feasible, and birth cohort effects were not possible to assess in this study.

## Conclusion

The diabetic population is on the rise. Costs of healthcare for the diabetic elderly are projected to have a growing share of total healthcare costs to the system in Costa Rica. Risk factors and behavioral differences between the diabetic and the non-diabetic population should be further studied. Obesity reduction and physical activity promotion must be taken into account in the formulation of public policies for diabetes prevention in this country. The challenge for public prevention policies is to develop and evaluate ways of addressing the underlying factors that make individuals vulnerable to the condition. Diabetes prevention, or at least delaying its onset, are possible. This would reduce the impact of the diabetic epidemic in the Costa Rican elderly population.

Health is not exclusively determined by individual choice; it is rather a process mediated by social determinants. Biological characteristics, but also lifestyle, human environment, health systems and policies all have an impact on human health. Type 2 diabetes, more than just a disease, is a result of social and cultural dynamics that can be modulated by appropriate policies.

Although problematic, having common risk factors for a number of metabolic conditions, including diabetes, is also a window of opportunity. Lifestyles are modifiable and although this is clearly not an easy task, public policies should redirect efforts towards population behavioral modifications. Controlling population nutritional status with strategies intervening not only with individuals but also with their environments would bring the benefits of preventing more than one condition while also simultaneously reducing costs.

The most highly effective interventions to reduce morbidity, premature mortality, and the incidence of complications that derive from diabetes are education for lifestyle change and the creation of environments in which individual behavioral initiatives can succeed. As stated by Yach et al. [58] overweight and obesity have become to diabetes what tobacco is to lung cancer. Acting on diabetes preventable risk factors is therefore mandatory to slow down diabetes incidence. Efforts to impact the quantity and quality of life of incoming cohorts should focus on primary healthcare services including extramural activities that go beyond medical consultations. Health promotion and prevention must be encouraged as strategies to act on diabetes risk factors in order to improve elderly population health.

## Data Availability

The datasets used during the current study are publicly available from http://creles.berkeley.edu:1313/CRdata.pl. Other materials may be available from the corresponding author on reasonable request.
